# Agnodice: indexing experimentally supported bacterial sRNA-RNA interactions

**DOI:** 10.1128/mbio.03010-23

**Published:** 2024-02-06

**Authors:** Vasiliki Kotsira, Giorgos Skoufos, Athanasios Alexiou, Maria Zioga, Spyros Tastsoglou, Filippos S. Kardaras, Nikos Perdikopanis, Zacharopoulou Elissavet, Vasileios Gouzouasis, Theodosia Charitou, Artemis G. Hatzigeorgiou

**Affiliations:** 1 DIANA-Lab, Department of Computer Science and Biomedical Informatics, University of Thessaly, Lamia, Greece; 2 Hellenic Pasteur Institute, Athens, Greece; 3 Department of Informatics and Telecommunications, National and Kapodistrian University of Athens, Athens, Greece; 4 Department of Molecular Biology and Genetics, Democritus University of Thrace, Alexandroupolis, Greece; 5 Department of Computer Science and Biomedical Informatics, University of Thessaly, Lamia, Greece; University of Delaware, Newark, Delaware, USA

**Keywords:** small RNAs, bacteria, interactions, RNA regulation, microbiology

## Abstract

In the last decade, the immense growth in the field of bacterial small RNAs (sRNAs), along with the biotechnological breakthroughs in Deep Sequencing permitted the deeper understanding of sRNA-RNA interactions. However, microbiology is currently lacking a thoroughly curated collection of this rapidly expanding universe. We present Agnodice (https://dianalab.e-ce.uth.gr/agnodice), our effort to systematically catalog and annotate experimentally supported bacterial sRNA-RNA interactions. Agnodice, for the first time, incorporates thousands of bacterial sRNA-RNA interactions derived from a diverse set of experimental methodologies including state-of-the-art Deep Sequencing interactome identification techniques. It comprises 39,600 entries which are annotated at strain-level resolution and pertain to 399 sRNAs and 12,137 target RNAs identified in 71 bacterial strains. The database content is exclusively experimentally supported, incorporating interactions derived via low yield as well as state-of-the-art high-throughput methods. The entire content of the database is freely accessible and can be directly downloaded for further analysis. Agnodice will serve as a valuable source, enabling microbiologists to form novel hypotheses, design/identify novel sRNA-based drug targets, and explore the therapeutic potential of microbiomes from the perspective of small regulatory RNAs.

**IMPORTANCE:**

Agnodice (https://dianalab.e-ce.uth.gr/agnodice) is an effort to systematically catalog and annotate experimentally supported bacterial small RNA (sRNA)-RNA interactions. Agnodice, for the first time, incorporates thousands of bacterial sRNA-RNA interactions derived from a diverse set of experimental methodologies including state-of-the-art Next Generation Sequencing interactome identification techniques.

## INTRODUCTION

Small RNAs (sRNAs) are predominantly non-protein-coding RNAs that are encountered in a wide range of eukaryotic and prokaryotic organisms and are involved in various biological functions with a prominent role in post-transcriptional regulation of gene expression. In the bacterial kingdom, sRNAs typically range between 50 and 500 nucleotides in length (1). Post-transcriptional regulation by bacterial sRNAs can have a positive or negative effect and is usually exerted via imperfect base-pairing with their target RNAs, in response to stress, metabolism, and environmental stimuli (2–4). Typically, bacterial sRNAs are divided into two broad categories, namely, *cis*-acting (antisense RNAs, asRNAs) and *trans*-acting RNAs (taRNAs). asRNAs are transcribed from the opposite strand of their targets and exert their regulatory role via extensive complementarity. On the contrary, taRNAs, the most well-studied bacterial sRNAs, are usually localized distally from their targets and exhibit limited complementarity with them (5). Bacterial sRNAs play major roles during bacterial infections (6, 7), in the control of antibiotic resistance genes and of virulence factors (8–11), during intra- and inter-species communication (12–14) and in other crucial molecular and cellular processes, deeming them extremely important for (i) basic research in microbiology, (ii) applied biomedical and clinical research, and (iii) the development of novel sRNA-based therapeutic interventions and biomarkers.

It’s only recently that the field of bacterial sRNAs has grown remarkably. Α flourishing body of evidence emerges, contributing to a more profound comprehension of the significance of bacterial sRNAs. Notably, advancements in Deep Sequencing techniques have facilitated the systematic detection of interactions between various sRNAs and RNAs in a high-throughput manner (15–24). To date, many experimental methods featuring diverse protocols, yet ultimately serving the same scope, have been developed. In RIL-Seq (RNA interaction by ligation and sequencing) (15, 25), CLASH (crosslinking, ligation, and sequencing of hybrids) and GRIL-Seq (global sRNA target identification by ligation and sequencing), interacting RNAs are ligated prior to sequencing enabling the generation of chimeric RNA fragments. While RIL-Seq and CLASH are RNA-binding protein (RBP)-specific (i.e., they rely on immunoprecipitation of a specific RBP of interest) and sRNA-agnostic (i.e., many sRNAs and many RNA targets are captured in one experiment), GRIL-Seq is RBP-agnostic and sRNA-specific (i.e., one sRNA of interest and multiple of its RNA targets are captured in one experiment, irrespective of the RBPs involved). However, it is important to note that RNA-RNA interactions derived from Deep Sequencing techniques may come with the potential risk of false positive results. While these methods provide valuable insights, the sensitivity and depth of sequencing can sometimes lead to the inclusion of spurious interactions, requiring careful validation and data interpretation.

**Fig 1:**
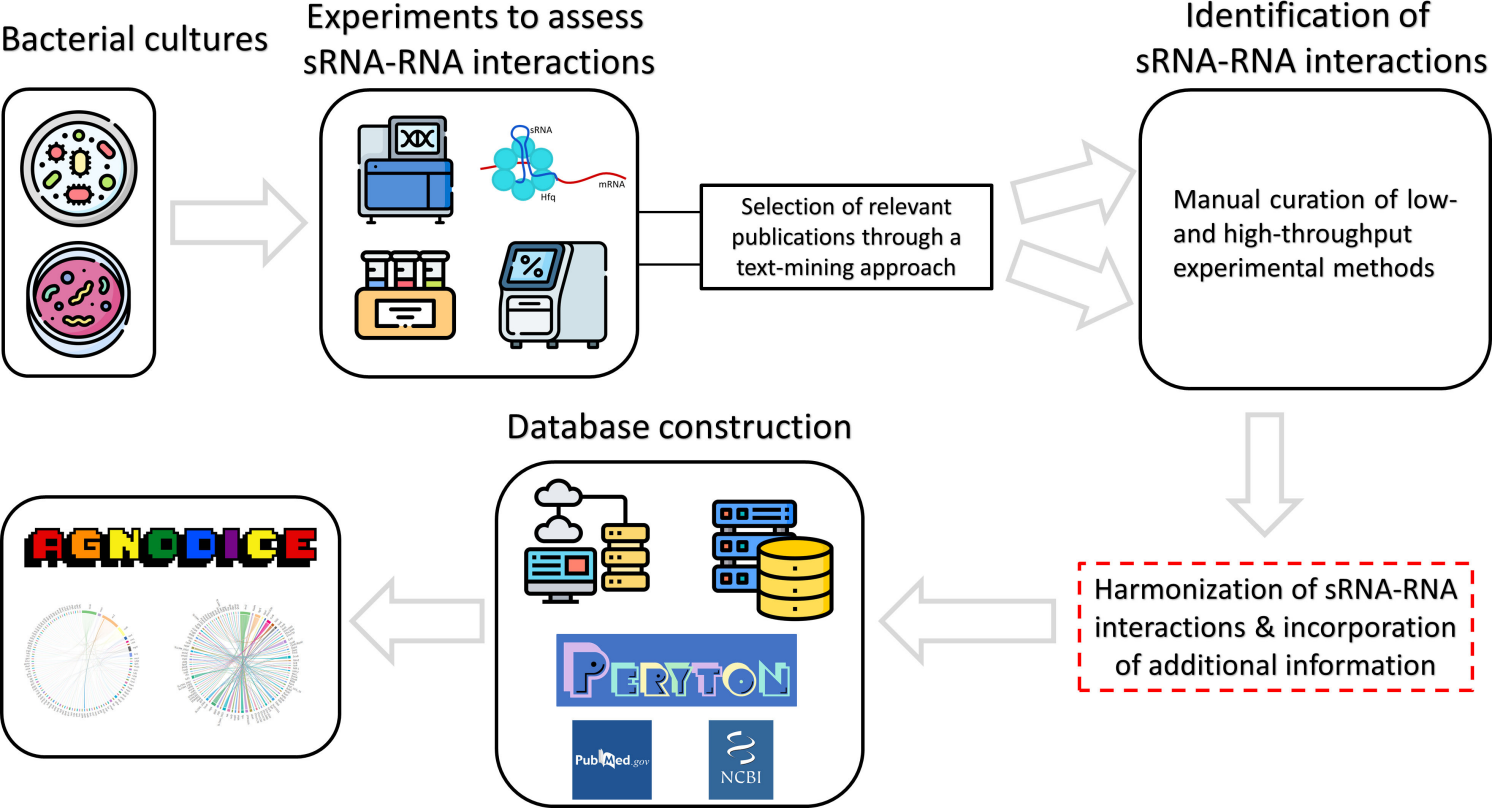
Schematic representation of the development and information flow, from the researcher bench to the creation of Agnodice resource. sRNA interactome data, generated by low- or high-yield experimental methods, are contained in hundreds of articles and Supplementary Materials. After querying for candidate sources, meticulous curation is applied. sRNA-RNA interactions are detected by means of manual curation. The resulting set of entries is harmonized, accompanied with a rich experiment- or study-specific metainformation and broken down into an efficient database schema. Inter-connection with external resources, including PubMed and NCBI Taxonomy and Peryton database of microbe-disease associations, is performed. The resulting content is provided in the form of an open, user-friendly online database, supporting numerous querying, filtering, visualization, and download functionalities.

Figure 1 presents an overview schema of Agnodice, our meticulous effort to record and curate experimentally supported bacterial sRNA-RNA interactions offered, for the first time, as a comprehensive collection of thousands of such events derived from a diverse set of interactome identification techniques, including cutting-edge Deep Sequencing methods. The arsenal of experimental techniques that are currently part of the database is divided into two broad categories; low- and high-throughput methods. These two categories are further subdivided into direct techniques (demonstrating RNA-RNA interactions in action) and indirect techniques (revealing an effect of the presence/absence of sRNAs on target RNAs, yet lacking direct evidence on RNA-RNA interactions). Agnodice interactions by both low- and high-throughput methods are exclusively derived through manual curation of the available literature.

## MATERIALS AND METHODS

### Data collection and curation process

In order to comprehensively collect interactomic Deep Sequencing data sets, we conducted systematic searches on PubMed using a predefined set of keywords. These keywords included terms such as “RIL-Seq,” “GRIL,” “CLASH,” “MAPS,” as well as combinations of “bacteria” and/or “sRNAs” and/or “interactions. This approach yielded a total of 24 data sets encompassing various Deep Sequencing methodologies, including LIGR-Seq, RIL-Seq, MAP-Seq, GRIL-Seq, rGRIL-Seq, and CLASH. Furthermore, in order to retrieve studies with experimentally confirmed interactions, we developed a text mining pipeline with full-text capacity dedicated to the identification of studies comprising sRNA-RNA interactions. For pre-processing, we used a set of approximately 200, manually selected, relevant scientific publications. Then, we evaluated every related full-text article, regarding the existence of sRNAs and targets, as well as by the importance of individual words on it. Sentences possibly containing the desired associations were retained for manual curation. Curators performed meticulous manual curation of studies potentially containing interactions verified by low-throughput methods, populating a table with 50 pre-defined fields. The final collection was independently cross-checked and post-processed to ensure the uniformity and quality of its content. The criteria required to record an interaction entry were as follows: (i) the interaction had to be rigorously supported by at least one experimental method and, for example, not be supported solely via means of computational target prediction, (ii) detailed information on the experimental design, microorganism, and interaction components should be provided by the authors, and (iii) interactions which were assessed using statistical methods (mainly high-throughput studies) should be deemed statistically significant (i.e., *P* < 0.05). Through the manual curation of low-yield methodologies, a total of approximately 800 interactions pertaining to 69 different bacterial strains were derived. To annotate and uniformly process interactions derived from high-throughput experiments, custom-made pipelines were constructed using the Python programming language. The scripts have been deposited in a public repository, accessible through the following link (GitHub link). Supplementary files from each study, along with annotation files obtained from NCBI and BioCyc (26) databases for each species, were utilized to populate each of the 50 fields in the database.

### Database architecture and implementation

Agnodice was built using the Model–View–Controller architecture as a relational database and is being hosted on Apache HTTP server 2.4. The backend consists of a PostgreSQL server 11.8 (https://www.postgresql.org/) where Agnodice’s data are stored in multiple tables featuring relational connections for optimal storing and querying. The PHP framework Laravel 8 (https://laravel.com/) (PHP 7.2) handles the backend logic including the connection to the PostgreSQL server for the storing and retrieval of the data. The front end is designed as a one-page website using Angular 14 (https://angular.io/) and the Angular Material UI library (https://material.angular.io/). Finally, the database statistics are presented using the Chart JS (https://www.chartjs.org/) library, while Flourish (https://flourish.studio/) is utilized for the more complex visualizations provided.

## RESULTS

### Database statistics and content

**Fig 2:**
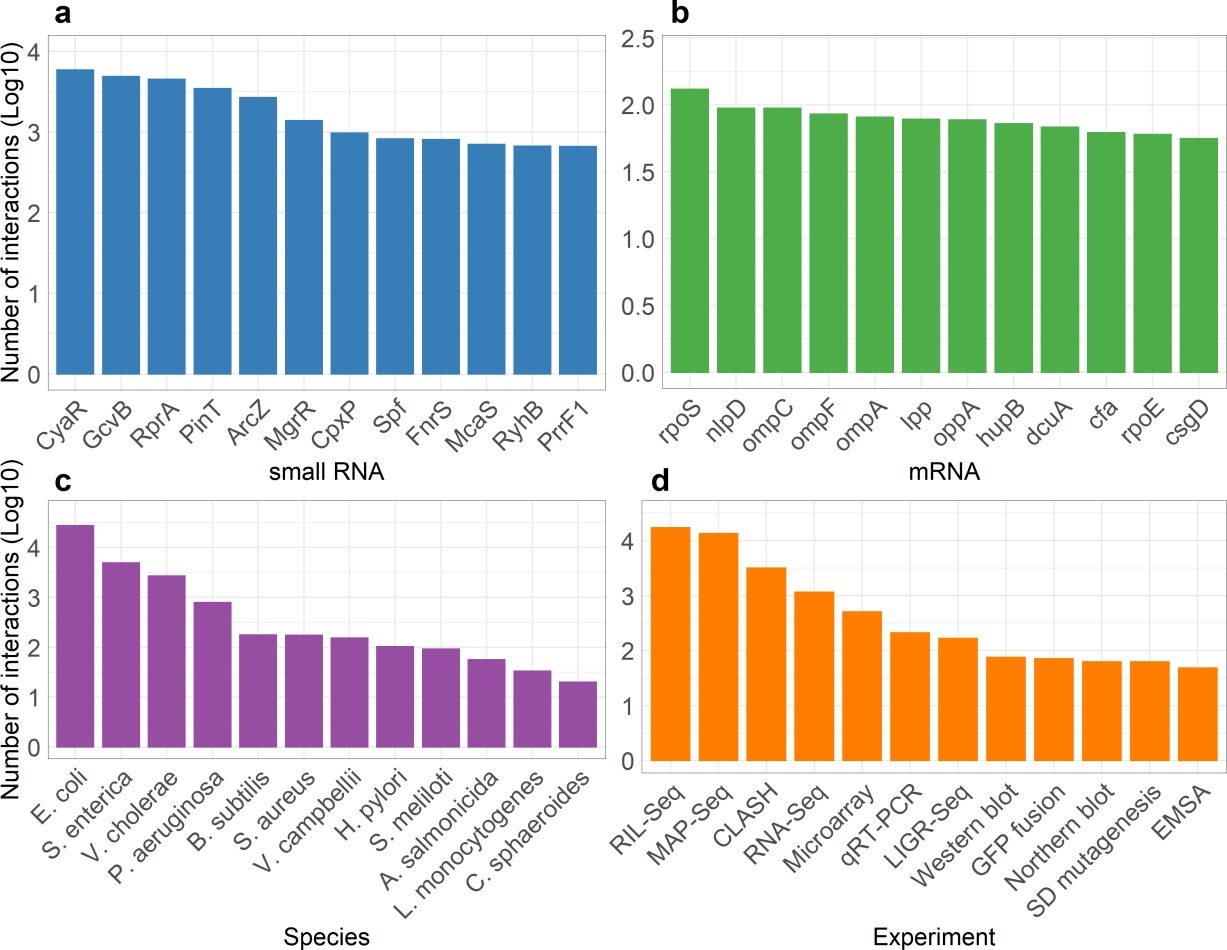
(a) Top 12 most frequent sRNA regulators and (b) regulated genes featured in Agnodice. (c) Top eight species in terms of total interactions in the database. (d) Number of total interactions per experimental method (interactions by low-yield methods summed together). Interaction sums are transformed in log10 space. As expected, these numbers are potentially skewed toward sRNAs originating from organisms that are extensively studied (e.g., *E. coli*).

Manual curation of more than 112 studies yielded 39,600 sRNA-RNA interactions. Agnodice database provides a total of 26,361 unique sRNA-RNA interacting pairs between 399 sRNAs and 12,137 coding/non-coding RNAs. Out of the total entries, more than 22,900 are derived from high-confidence methodologies directly assessing RNA-RNA binding sites. In addition, Agnodice incorporates 10,490 coding RNAs annotated with ~7,257 unique RNA products (i.e., proteins) among included strains, and 917 non-coding RNAs. In total, Agnodice features interactions derived from 37 experimental methods (28 low- and 9 high-throughput methods). The database comprises interactions dependent on three different RBPs, the major regulator Hfq, CsrA, and ProQ, as well as interactions for which no RBP-related evidence was obtained. For every annotated interaction, the database integrates additional information including article metadata, microorganism names and TaxIDs (exclusively derived from NCBI Taxonomy database), bacterial lineages (i.e., phylum, genus, species, strain names), information on the specifics of the applied experimental methods, and more. Additionally, Agnodice provides details about the sRNA, including synonym names, coordinates, sequence, upstream and downstream flanking genes, and strand information. Finally, predicted pairings between sRNAs and RNA molecules are provided for ~70% of the interactions hosted in the database. These pairings were generated using the IntaRNA 2.0 algorithm (27), a program designed for the rapid and accurate prediction of interactions between two RNA molecules. IntaRNA was exclusively employed for interactions where sRNAs were annotated through NCBI. Basic statistics of Agnodice are presented in Fig. 2. Additionally, Fig. S1 to S4 comprise upset plots that depict the number of genes commonly and distinctly regulated by sRNAs in the species *Escherichia coli*, *Salmonella enterica*, *Pseudomonas aeruginosa*, and *Vibrio cholerae*.

### Comparison with existing resources

Databases addressing similar topics include *RegulonDB* (28), *sRNAMap* (29), *sRNAdb* (30), *BSRD* (31), and *sRNATarBase* (32)*. RegulonDB*, hosting ~230 sRNA-mRNA interactions, is a reference database dedicated to the species *E. coli K-12*. sRNAMap emphasizes sRNA annotation and provides ~60 sRNA interactions. sRNAdb also focuses on bacterial sRNA annotations, lacking information on their targets. BSRD provides ~205 validated sRNA-RNA interactions. Finally, sRNATarBase v3.0 features ~500 sRNA-RNA interactions that are experimentally supported by low-throughput methods.

In addition, one recently published study provides an easily accessible and interactive browser where users can navigate through bacterial sRNA interactions, which have exclusively been derived through RIL-Seq data sets. The browser stores built-in RIL-Seq-derived interactions from previously conducted experiments but also offers to the users the option to upload and visualize their own RIL-Seq results (33). Finally, targets with experimental support may also be found in another online resource, that provides RNA-RNA interactions (34) derived exclusively from two RIL-Seq experiments.

Agnodice incorporates by far the largest number (39,600) of experimentally supported bacterial sRNA-RNA entries. It is unique in (i) supporting advanced querying/filtering capacity in an intuitive user-friendly scheme and (ii) hosting interactions derived from a diverse set of low-yield and state-of-the-art Deep Sequencing interactome identification techniques (e.g., RIL-Seq, CLASH, MAPS, LIGR-Seq).

### Database functionality and interface

**Fig 3:**
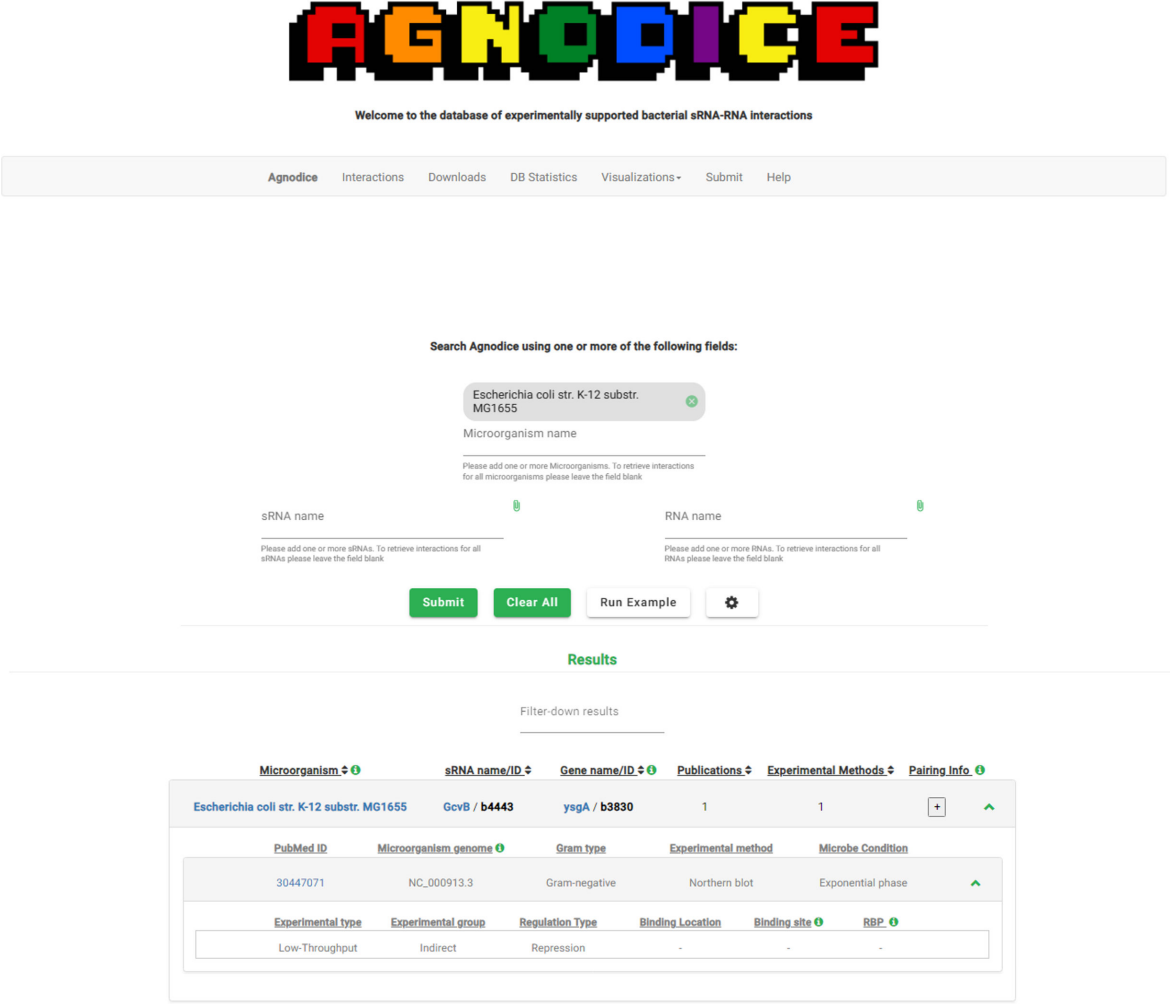
Demonstration of the results offered through the user interface of Agnodice as generated by an example query. Basic information is given in a first-level tabular format, while supplemental information regarding each initial entry can be disclosed through specialized buttons that allow users to reveal the corresponding sub-tables hosting it.

Agnodice is built under a mindset of a user-friendly interface that provides researchers with a variety of functionalities and allows them to reach out to an all-in-one resource, perform hypotheses, come up with potentially interacting RNA candidates, and unravel complex biological questions. The database supports queries using one or more sRNA(s), gene(s), and/or microorganism names from any genus, species, and strain taxonomic ranks, as well as the application of smart filtering options, including “regulation type” (i.e., activation, repression, or unknown), “experimental method name,” “publication year,” etc. Furthermore, filtering options incorporate a check box for including only interactions derived using Deep Sequencing-based techniques directly assessing RNA-RNA binding events. Also, special effort has been placed to offer direct interconnections with a number of useful resources including NCBI Taxonomy (35) reference organism indices, PubMed database to access relevant publications, and Peryton (36), our state-of-the-art database of experimentally supported microbe-disease associations. An example of the results offered to the database’s interface following a user query is presented in Fig. 3. Furthermore, a dedicated “Downloads” page has been developed in which users can download the entire collection of interactions without the need for a query and/or filtering. Through a dedicated Visualizations page, users can utilize Chord diagrams to focus on sRNA-RNA interactions that have been independently reproduced by at least two different studies in the species *E. coli*, *S. enterica*, and *P. aeruginosa*. The DB Statistics page presents the top 12 entries among key components of Agnodice (namely, microorganisms, sRNAs, genes, and experimental methods). Unrestricted download options with additional complementary metadata (e.g., publication information, bacterial lineage details) permit the storage of Agnodice results locally, allowing users to incorporate them in any meta-analysis scenario of their own. A detailed and informative Help page is available to ensure that users perform tasks across our resources in an effortless way. Interested researchers can submit their own interactions through the web interface, specifically for new publications that are not yet part of the Agnodice database. These submissions will be carefully examined and manually inspected by our curators using the same methodology that has been applied to all interactions within the database. During this curation process, the submitted interactions will remain in a provisional state until they have been reviewed and verified. By following this approach, we aim to maintain the highest standards of data quality and consistency in our database. We encourage the scientific community to actively contribute to the future establishment of a centralized information hub for bacterial sRNA-RNA interactions. These collaborative efforts will facilitate the initiation of new experiments and promote the advancement of our collective knowledge in this field.

## DISCUSSION

Agnodice constitutes the first comprehensive database of experimentally supported bacterial sRNA-RNA interactions, currently hosting the largest number of interactions (39,600), as evidenced by a wide assortment of methodologies. Today, a plethora of research projects and laboratories are pushing the envelope of bacterial small RNA research by actively pursuing and realizing novel experimental and computational techniques (33, 37–44). Breakthrough works from the last few years demonstrate not only the importance of bacterial sRNAs but also their beauty and complexity. Intriguingly, bacterial small RNAs have been recently shown to participate in *trans*-kingdom RNA-RNA interactions (14) with their hosts’ protein-coding genes, adding an extra dimension of complexity but also a new, extremely interesting and important research topic. We are determined that Agnodice will function as a reference map of notable bacterial small RNA targeting events, facilitating their further experimental scrutiny to better apprehend bacterial RNA regulation and exploit its potential therapeutic and biomarker value.

## SUPPLEMENTAL MATERIAL

Supplemental Figures mbio.03010-23-s0001.docx Figures S1 to S4.

ASM does not own the copyrights to Supplemental Material that may be linked to, or accessed through, an article. The authors have granted ASM a non-exclusive, world-wide license to publish the Supplemental Material files. Please contact the corresponding author directly for reuse.
