# Supplementary material for: Agnodice: indexing experimentally supported bacterial sRNA-RNA interactions
Source: mBio. 2024 Feb 6;15(3):e03010-23. doi: 10.1128/mbio.03010-23 (PMC10936433; doi:10.1128/mbio.03010-23)
Supplement: Supplemental Figures — Figures S1 to S4. [file mbio.03010-23-s0001.docx]

**Agnodice: indexing experimentally supported bacterial sRNA-RNA interactions**

**Supplementary material**

**
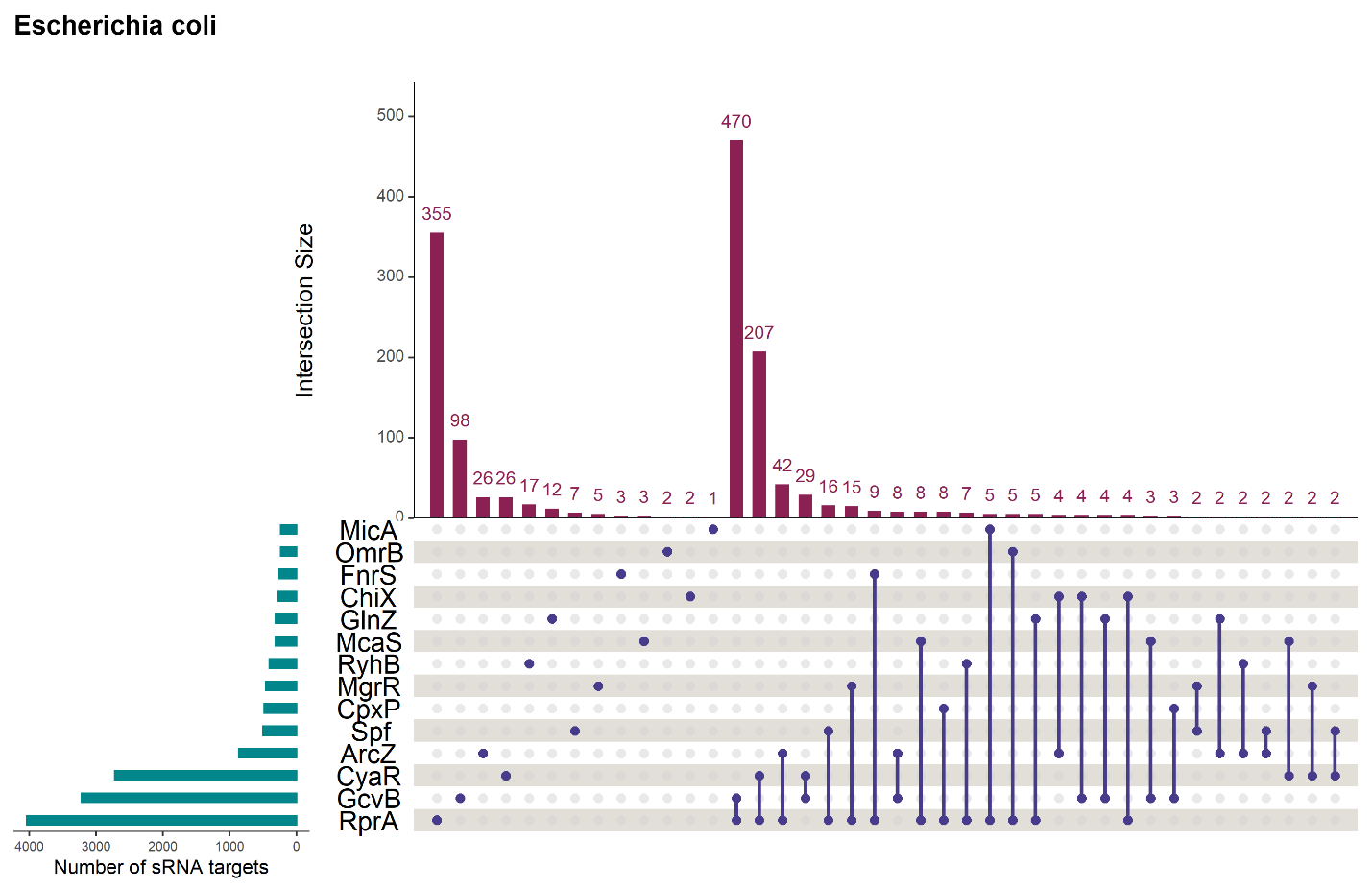
**

**Figure 1**: Upset plot depicting the number of commonly and distinctly number of genes regulated by E. coli sRNAs. Only sRNAs with at least 300 targets are participating in the plot.


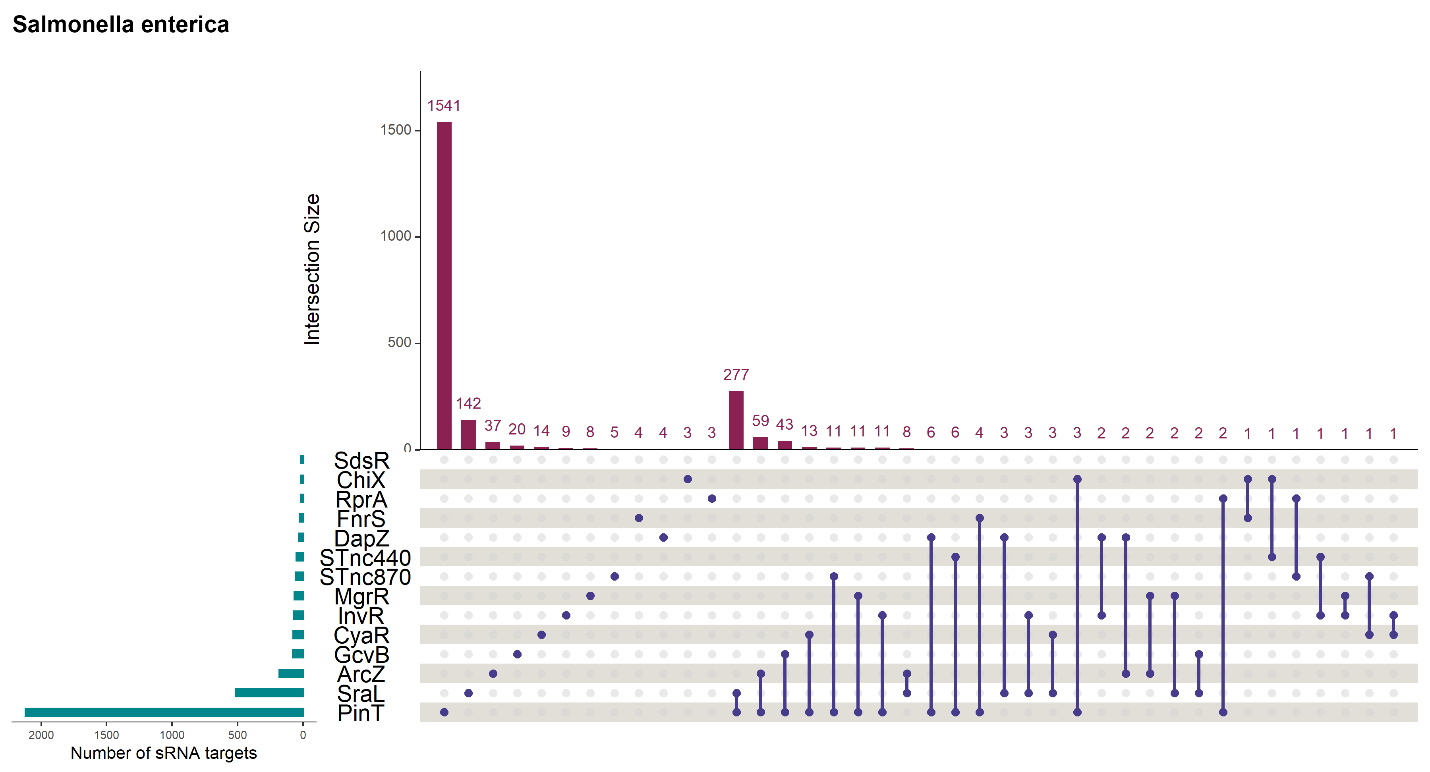


**Figure 2**: Upset plot depicting the number of commonly and distinctly number of genes regulated by S. enterica sRNAs. Only sRNAs with at least 20 targets are participating in the plot.


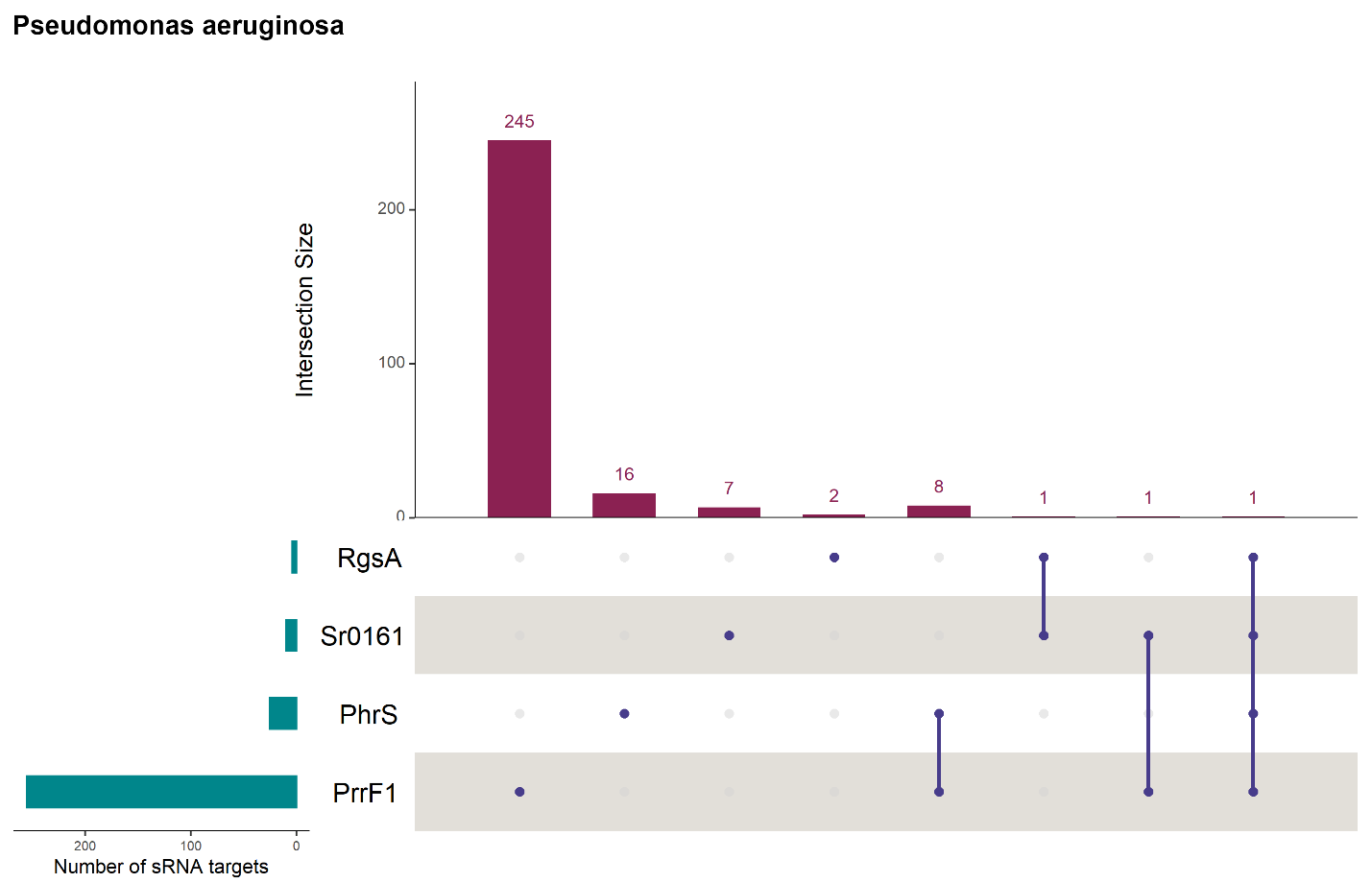


**Figure 3**: Upset plot depicting the number of commonly and distinctly number of genes regulated by P. aeruginosa sRNAs. Only sRNAs with at least 20 targets are participating in the plot.


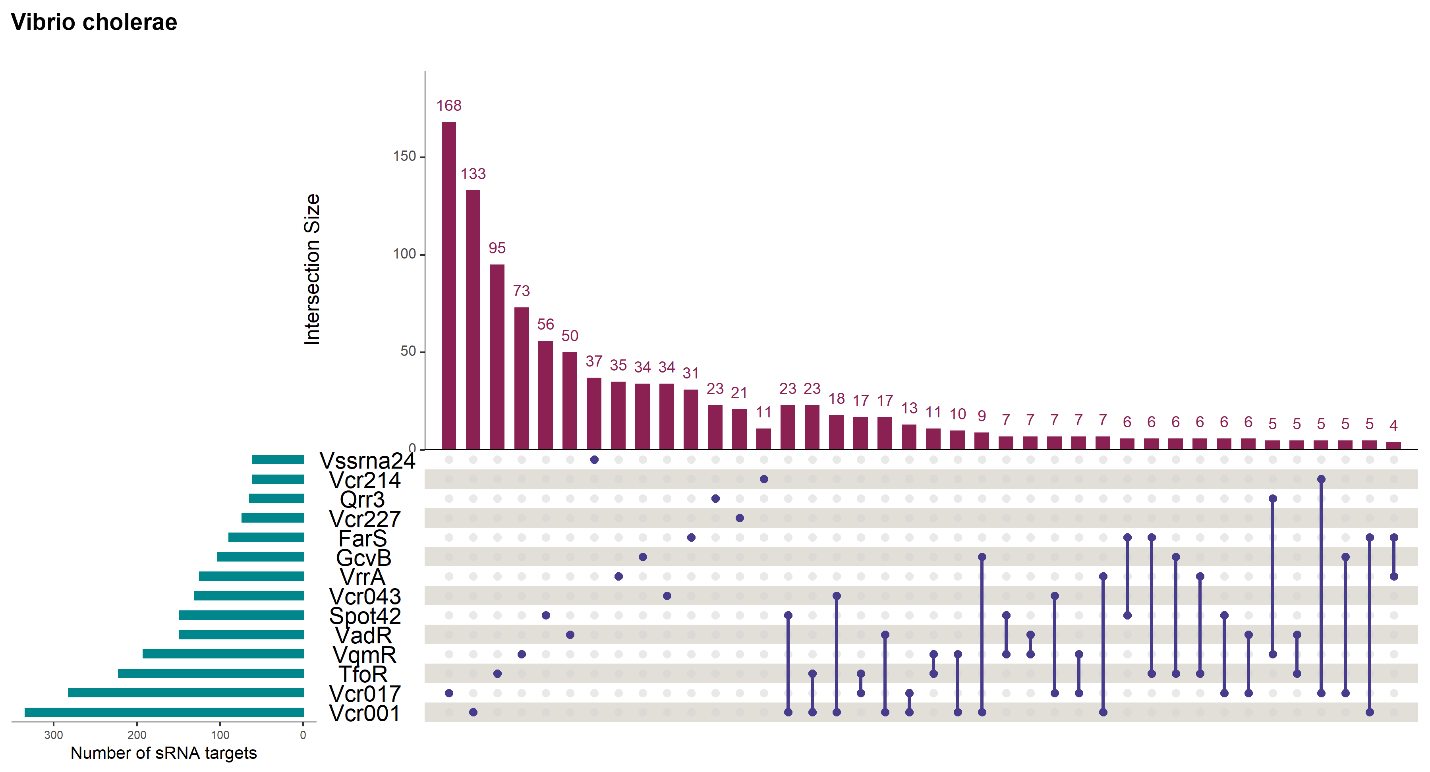


**Figure 4**: Upset plot depicting the number of commonly and distinctly number of genes regulated by V. cholerae sRNAs. Only sRNAs with at least 20 targets are participating in the plot.
